# Therapeutic Potential of PARP Inhibitors in the Treatment of Metastatic Castration-Resistant Prostate Cancer

**DOI:** 10.3390/cancers12113467

**Published:** 2020-11-21

**Authors:** Albert Jang, Oliver Sartor, Pedro C. Barata, Channing J. Paller

**Affiliations:** 1 Deming Department of Medicine, Hematology-Oncology Section, Tulane University School of Medicine, New Orleans, LA 70112, USA; ajang@tulane.edu (A.J.); osartor@tulane.edu (O.S.); pbarata@tulane.edu (P.C.B.); 2 Tulane Cancer Center, New Orleans, LA 70112, USA; 3 The Sidney Kimmel Comprehensive Cancer Center at Johns Hopkins, Johns Hopkins University School of Medicine, Baltimore, MD 21231, USA

**Keywords:** metastatic castration-resistant prostate cancer, PARP inhibitor, olaparib, rucaparib, niraparib, talazoparib

## Abstract

**Simple Summary:**

In recent years, the development of sequencing techniques to reveal the genomic information of prostate cancer tumors has allowed for the emergence of targeted therapies. Genomic aberrations in tumor cells have become popular due to the successful development of PARP inhibitors, which are particularly active in those tumors harboring DNA repair genomic defects. This review focuses on PARP inhibitors, two of which were approved for use by the US Food and Drug Administration in 2020 in metastatic castration-resistant prostate cancer. The article highlights the development of PARP inhibitors in the preclinical setting, summarizes the impactful clinical trials in the field, and discusses the need for continued research for further success in treating men with advanced prostate cancer.

**Abstract:**

Metastatic castration-resistant prostate cancer (mCRPC) is an incurable malignancy with a poor prognosis. Up to 30% of patients with mCRPC have mutations in homologous recombination repair (HRR) genes. Poly (ADP-ribose) polymerase (PARP) inhibitors take advantage of HRR deficiency to kill tumor cells based on the concept of synthetic lethality. Several PARP inhibitors (PARPis) have been successful in various malignancies with HRR gene mutations including BRCA1/2, especially in breast cancer and ovarian cancer. More recently, olaparib and rucaparib were approved for mCRPC refractory to novel hormonal therapies, and other PARPis will likely follow. This article highlights the mechanism of action of PARPis at the cellular level, the preclinical data regarding a proposed mechanism of action and the effectiveness of PARPis in cancer cell lines and animal models. The article expands on the clinical development of PARPis in mCRPC, discusses potential biomarkers that may predict successful tumor control, and summarizes present and future clinical research on PARPis in the metastatic disease landscape.

## 1. Introduction

Metastatic castration-resistant prostate cancer (mCRPC) patients include men with distant metastases who have evidence of disease progression defined by either prostate-specific antigen (PSA) progression, new metastases, or clinical symptoms, despite a castrate level of testosterone [1]. While the success of androgen deprivation therapy (ADT) in suppressing tumor progression is almost universal, most patients invariably progress to castration-resistant prostate cancer. Exact mechanisms of progression to castration resistance remain unclear [2,3]. The current best hypothesis is the presence of a sustained androgen receptor signal, with several possible mechanisms leading up to this [3]. Although several therapeutic options have been developed for mCRPC, it remains incurable with a poor prognosis, with median survival for patients with mCRPC of approximately two to three years [4,5,6].

Analysis of whole-exome sequencing for mCRPC tumors has revealed a complex genomic landscape with variability among patients, but up to two-thirds of patients are found with genomic alterations in non-androgen-receptor-related pathways as possible therapeutic targets [2,7]. Germline and somatic mutations in homologous recombination repair (HRR) genes (including *BRCA1*, *BRCA2*, *ATM*, *CHEK2* and others—expanded further in the following section) exist in 15–30% of cases, which increase in frequency during later stages of the disease [2,8,9,10]. Based on germline studies conducted in men with metastatic disease, DNA repair alterations are expected in approximately 12–17% of the time, more often including *BRCA2*, *ATM*, and *CHEK2* genes, which is significantly higher than the incidence of men with localized prostate cancer or with family history of prostate cancer [8,11]. The high prevalence of germline mutations has led to the National Comprehensive Cancer Network recommendation of germline testing in all patients with metastatic disease [12]. However, clinical implications of germline and somatic HRR gene alterations, besides germline *BRCA2* mutations, in prostate cancer remain uncertain due to limited studies, number of patients enrolled, and available approved treatments [13].

One actionable key protein involved in deoxyribonucleic acid (DNA) repair machinery is poly (ADP-ribose) polymerase (PARP). Targeted therapies against DNA repair proteins include the use of PARP inhibitors (PARPis), and the clinical development of these drugs has recently expanded to multiple tumor types. This article provides an overview of the mechanism of action and rationale for the use of PARPis alone or in combination with other therapies with an emphasis on studies leading to approval for use for mCRPC. The article summarizes the current data on putative predictive biomarkers and discusses present and future clinical trials with PARPis as they relate to mCRPC.

## 2. The Role of Cellular DNA Repair and Poly (ADP-Ribose) Polymerase

A complex system to repair DNA damage is in place to amend errors before cells replicate [14]. Major DNA repair pathways include direct repair, mismatch repair, base excision repair, nucleotide excision repair, and double-strand break recombination repair (which includes non-homologous end-joining and HRR) [15]. PARPs are nuclear enzymes involved in the enzymatic machinery for repairing DNA breaks, and in other roles including gene regulation, cell proliferation and cell death [16,17]. Since the original discovery of PARP-1 in 1963 [18], at least 18 PARPs have been identified [17].

PARPs attach poly (ADP-ribose) polymers to proteins, including to one another and to themselves with the ability to self-modify. When DNA becomes damaged, PARP-1 is recruited to the site of single-strand breaks, and it begins to cleave nicotinamide adenine dinucleotide (NAD+) into nicotinamide and ADP-ribose. PARP-1 catalyzes formation of ADP-ribose polymers (PARylation), which helps release the DNA from histones, and it also recruits and activates the base excision repair (BER) enzyme complex [19]. PARP-1 and PARP-2 both promote BER by two independent but intrinsically linked mechanisms, and disruption of both proteins leads to defective BER [20]. When BER is defective, homologous recombination (HR) may be able to rescue the damaged DNA and ensure proper DNA replication. HR is one of the most effective mechanisms to repair double-strand DNA breaks, which requires functional BRCA1 and BRCA2 proteins [21]. Other HR genes identified include *PALB2*, *FANCI*, *FANCL*, *FANCC*, *RAD50*, *RAD51*, *RAD51C*, *RAD54L*, *ATM*, *ATR*, *CHEK1*, and *CHEK2* [22].

PARP inhibitors (PARPis) appear to work in two different ways: they compete with NAD+ at the catalytic site of PARPs to prevent PARylation, and they also trap PARP1/2 to damaged DNA to form cytotoxic PARP-DNA complexes [23]. Different PARPis were found to have varying potency of trapping PARP-DNA complexes not related to their catalytic inhibitory properties. PARP inhibition leads to stalling of replication forks, inducing double-strand DNA breaks and the need for HR [24,25]. Tumor cells with two mutated *BRCA* genes are unable to start HR, which ultimately leads to cell death, while normal cells still possess at least one functioning *BRCA* gene to begin HR to repair DNA and prevent apoptosis. Cells that have either BER deficiency or HR deficiency may survive; if both pathways are deficient, the cells die. This is the concept for using PARPis to selectively target malignancies with *BRCA1/2* germline or somatic mutations, which is termed “synthetic lethality.” This model was supported by two landmark studies published in 2005–BRCA1/2 dysfunction sensitized tumor cells to PARPis in vitro, resulting in selective tumor cell death [26,27].

## 3. Preclinical Development of PARP Inhibitors

Olaparib (previously known as AZD2281 and KU-0059436) was discovered to be a potent orally bioavailable PARPi against *BRCA-1/2* deficient cell lines. It was determined to have both good in vitro cellular potency (IC_50_ for PARP-1 inhibition of 6 nM) and in vivo efficacy because it led to 80% tumor inhibition when fed in combination with temozolomide to mice [22]. Olaparib also had in vitro activity against ataxia telangiectasia mutated (*ATM*)-deficient lymphoid tumors and in vivo activity in mice xenografted with *ATM*-deficient mantle cell lymphoma tumor [28,29].

Rucaparib (PF01367338 and AG014699) was selected from a group of several compounds tested for a clinical trial based on excellent chemosensitization, radiosensitization, aqueous solubility, and safety profile [30]. Rucaparib was cytotoxic to human cancer cell lines with mutated *BRCA1/2* and was then found to be effective in a wide range of ovarian cancer cell lines (including homologous recombination pathway deficiencies not including *BRCA1/2* mutations) alone and in combination with other agents [31,32].

Similarly, niraparib (MK-4827) [33], talazoparib (MDV3800 and BMN-673) [34], and veliparib (ABT-888) [35] moved forward to clinical trials based on potent in vitro activity against PARPs and successful oral bioavailability and in vivo activity in BRCA-deficient xenograft tumor murine models. Talazoparib was the most potent PARPi in vitro, about 100-fold more potent at trapping PARP-DNA complexes compared to both olaparib and rucaparib [36].

Research has shed some light on how PARPs are involved in prostate cancer and how PARPis are beneficial against prostate cancer, although the available literature is scant. The erythroblast transformation specific (ETS) gene fusion family is involved in the progression of a variety of cancers, and *TMPRSS2:ERG* is a prostate cancer-specific gene fusion; it has been noted that this gene fusion product interacts with PARP-1, and that olaparib was able to suppress ETS-positive (but not ETS-negative) prostate cancer cell invasion [37], yet no clinical data confirming this cellular effect is available. In fact, in a clinical trial with a subset of mCRPC patients who received veliparib and abiraterone, there was no difference in response rate between patients with or without ETS fusions [38]. The combination of rucaparib with radiation therapy was synergistic for prostate cancer cells expressing the *TMPRSS2:ERG* gene fusion, as these cells showed enhanced sensitivity towards rucaparib, which increased radiation response [39]. Schiewer et al. [40] demonstrated in prostate cancer cells that PARP-1 modulated both androgen-receptor function and response to DNA damage, suggesting its involvement in prostate cancer progression and maintenance of castration resistance; in this study, olaparib and veliparib successfully decreased androgen-receptor target gene expression and tumor growth using murine models and ex vivo prostate tumor cultures.

## 4. Clinical Development of Olaparib and Rucaparib in Prostate Cancer

### 4.1. Olaparib

The first clinical trial of olaparib (NCT00516373) included three patients with advanced prostate cancer, including one patient with a *BRCA2* mutation who had a greater than 50% reduction in PSA level and resolution of bone metastases [41]. The open-label phase II trial (NCT01078662) by Kaufman et al. [42] included eight patients with mCRPC (one with *BRCA1* mutation, seven with *BRCA2* mutation) and a median of two prior therapies. Median total duration of olaparib treatment was 223.5 days, given at 400 mg twice daily. The one *BRCA1* mutation patient and three of the seven *BRCA2* mutation patients responded to the treatment. Two of the eight prostate cancer patients had stable disease that persisted for at least eight weeks. Median duration of response was 327 days, and median time to onset of response was 54.5 days. Progression-free survival (PFS) at 6 months was 62.5%, median overall survival was 18.4 months, and 50.0% of the patients were alive at 12 months.

Further encouraging efficacy data was reported in the open-label Phase II Trial of Olaparib in Patients with Advanced Castration Resistant Prostate Cancer (TOPARP-A trial, NCT01682772), which focused solely on mCRPC [43]. The primary endpoint was based on objective response rate (ORR) according to Response Evaluation Criteria in Solid Tumors (RECIST), PSA decline, or reduction in circulating tumor-cell count. The trial enrolled 50 patients, although one was lost to follow-up early and was not included in the later analysis. All 49 patients had previously received treatment with other agents, including docetaxel, abiraterone, enzalutamide, and cabazitaxel. Next-generation sequencing data revealed that 16 patients had tumor aberrations in DNA-repair genes, including *BRCA1*, *BRCA2*, *ATM*, *FANCA*, *PALB2*, *HDAC2*, and *CHEK2*. Median duration of olaparib was 12 weeks. Patients with these DNA-repair gene aberrations had a significantly better response to olaparib of 400 mg twice daily (14/16 compared to 2/33 biomarker-negative patients). All seven patients with *BRCA2* loss had PSA levels decrease by over 50% from baseline, and the five who had measurable disease had radiologic partial response. Four of five patients with *ATM* mutations had response as well. The results in the TOPARP-A trial led the FDA in January 2016 to give olaparib a breakthrough therapy designation for mCRPC with *BRCA1/2* or *ATM* mutations previously treated with a taxane-based chemotherapy and either enzalutamide or abiraterone.

On a continuum, TOPARB-B was an open-label phase II trial that included 98 mCRPC patients with known aberrations to 18 DNA-repair genes who received olaparib: 49 received 300 mg twice daily and 49 received 400 mg twice daily [44]. Overall, 43 of the 98 patients achieved a confirmed composite response. Patients with the *BRCA1/2* mutation had the best response and longest median radiographic progression-free survival (rPFS) compared to patients with the 16 other gene mutations, but because olaparib did have an effect on these other mutations as well, this study supported the genomic stratification of mCRPC and olaparib’s potential in mCRPC patients with tumor gene aberrations besides the *BRCA1/2* mutation.

These data leveraged conducting a confirmatory, phase III trial, PROfound (NCT02987543). This prospective, randomized, open-label study evaluated the efficacy and safety of 300 mg of olaparib twice daily versus 160 mg of enzalutamide daily or 1000 mg of abiraterone daily in 387 patients with mCRPC and 15 HRR gene alterations (*BRCA1*, *BRCA2*, *ATM*, *BRIP1*, *BARD1*, *CDK12*, *CHEK1*, *CHEK2*, *FANCL*, *PALB2*, *PPP2R2A*, *RAD51B*, *RAD51C*, *RAD51D* or *RAD54L*) [45]. The patients had all been treated previously with enzalutamide or abiraterone, and some patients had also been treated previously with taxane chemotherapy. Cohort A included 245 patients (162 received olaparib and 83 received the control treatment) with at least one alteration in *BRCA1*, *BRCA2*, or *ATM,* while cohort B included 142 patients (94 received olaparib and 48 received the control treatment) with the 12 other gene alterations. The primary endpoint was imaging-based PFS in cohort A. In cohort A, the olaparib group was better than the control group in terms of significantly increased median imaging-based PFS (7.4 vs. 3.6 months, hazard ratio [HR] 0.34; 95% confidence interval [CI], 0.25 to 0.47; *p* < 0.001), median overall survival (18.5 vs. 15.1 months, HR 0.64; 95% CI, 0.43 to 0.97; *p* = 0.02), objective response rate (33% vs. 2%, odds ratio 20.86; 95% CI, 4.18 to 379.18; *p* < 0.001), and median time to pain (HR 0.44; 95% CI, 0.22 to 0.91; *p* = 0.02). For cohorts A and B together, the olaparib group was also better than the control group based on median imaging-based PFS (5.3 vs. 3.5 months, HR 0.49; 95% CI, 0.38 to 0.63; *p* < 0.001), confirmed objective response rate (22% vs. 4%, odds ratio, 5.93; 95% CI, 2.01 to 25.40), free of pain progression at six months (85% vs. 75%, HR 0.64), estimated median overall survival (17.5 vs. 14.3 months, HR 0.67; 95% CI, 0.49 to 0.93), and PSA_50_ response (30% vs. 10%).

For cohort A, the *ATM* group demonstrated limited activity (62 olaparib patients with median rPFS 5.36 months, 95% CI, 3.61 to 6.21 months; vs. 24 control patients with median rPFS of 4.70 months, 95% CI, 1.84 to 7.26 months), as further discussed in a later section. The *BRCA1* group had a small sample size (8 olaparib patients with median rPFS of 2.07 months, 95% CI, 1.38 to 5.52 months; vs. 5 control patients with median rPFS of 4.70 months, 95% CI, 1.71 to 3.71 months). For cohort B, interpreting results are limited by the relatively small sample size. Yet, promising findings were described in the *RAD51B* (4 olaparib patients with median rPFS of 10.89 months, 95% CI, 1.61 to 14.75; vs. 1 control patient with median rPFS of 1.77 months) and the *RAD54L* groups (3 olaparib patients with median rPFS of 7.20 months, 95% CI, 3.71 to 7.39; vs. 2 control patients with 2.41 months, 95% CI, 1.81 to 3.02 months).

In cohort A, more adverse events (AEs), including grade 3 or higher, were noted in the olaparib group compared to the control group in the PROfound trial, and in line with the known safety profile of PARP inhibitors [46,47]. While common AEs included anemia, nausea and decreased appetite, serious side effects associated with olaparib include the development of myelodysplastic syndrome, acute myeloid leukemia, and pneumonitis [48]. Thus, it is often helpful to obtain a complete blood count at baseline and then monthly to monitor for clinically significant changes.

This first biomarker-selected mCRPC study led the FDA in May 2020 to approve the use of olaparib for patients with mCRPC and HRR gene mutations who progressed despite previous treatment on enzalutamide or abiraterone [49]. Foundation-One was the approved companion diagnostic test, but other tissue and circulating tumor DNA assays are commercially available and future validation studies will clarify their role in identifying these biomarkers.

The efficacy of olaparib monotherapy in mCRPC patients without HRR mutations is under investigation. In a double-blinded, randomized, placebo-controlled phase II trial of mCRPC not required to have an HRR mutation (NCT0197221), 71 patients who received 300 mg of olaparib twice daily combined with 1000 mg of abiraterone daily were compared to 71 patients who received abiraterone and placebo [50]. Median rPFS was 13.8 months for olaparib and abiraterone compared to 8.2 months for abiraterone alone (HR 0.65, 95% CI, 0.44 to 0.97, *p* = 0.034), suggesting possible benefits for olaparib in mCRPC patients without HRR mutations. The ongoing phase III trial PROPEL (NCT03732820) builds on the success of the phase II trial that used the combination of olaparib and abiraterone in genomic unselected mCRPC patients. The primary outcome measure is rPFS, with expected completion in 2021.

### 4.2. Rucaparib

Rucaparib was granted accelerated approval by the FDA in May 2020 for treatment of mCRPC with a deleterious germline or somatic BRCA mutation previously treated with androgen-receptor-directed therapy and a taxane-based therapy, based on data from TRITON-2 [51]. The Trial of Rucaparib in Prostate Indications (TRITON)-2 is an open-label phase II trial (NCT02952534) evaluating 600 mg of rucaparib twice daily (with gonadotropin-releasing hormone [GnRH] analogue or prior bilateral orchiectomy) in mCRPC patients who progressed on androgen-deprivation therapy and one prior taxane-based chemotherapy with a deleterious germline or somatic alteration in *BRCA1*, *BRCA2*, *ATM*, *BARD1*, *BRIP1*, *CDK12*, *CHEK2*, *FANCA*, *NBN*, *PALB2*, *RAD51*, *RAD51B*, *RAD51C*, *RAD51D*, or *RAD54L*. Of 62 BRCA-mutated patients, 27 had a confirmed ORR, and 15 of these 27 had a response duration of at least six months. TRITON-3 is an actively recruiting, randomized, open-label phase III trial (NCT02975934) studying rucaparib 600 mg twice daily versus either abiraterone, enzalutamide, or docetaxel in patients with mCRPC and a deleterious germline or somatic mutation in *BRCA1*, *BRCA2*, or *ATM* that progressed on androgen-receptor signaling-directed therapy, building on the success of TRITON-2.

## 5. Other PARP Inhibitors in Prostate Cancer

Niraparib is being evaluated in the open-label phase II trial GALAHAD (NCT02854436) in mCRPC patients with gene alterations in *BRCA1*, *BRCA2*, *ATM*, *FANCA*, *PALB2*, *CHEK2*, *BRIP1*, or *HDAC2* who progressed despite androgen-receptor-targeted therapy and taxane-based chemotherapy [52]. At the 2019 European Society for Medical Oncology (ESMO) Congress, data presented on 81 patients (46 *BRCA* and 35 non-*BRCA*) showed better performance for *BRCA* patients, who had a 41% objective response rate, 63% complete response rate, median rPFS of 8.2 months, and overall survival of 12.6 months; for non-*BRCA* patients, the numbers were 9%, 16%, 5.3 months, and 14.0 months, respectively. This led the FDA in October 2019 to give niraparib a breakthrough designation as therapy for *BRCA1/2*-mutant positive mCRPC [53].

One milligram of talazoparib daily is being tested in the open-label phase II trial TALAPRO-1 (NCT03148795) in patients with mCRPC with mutations in *ATM*, *ATR*, *BRCA1*, *BRCA2*, *CHEK2*, *FANCA*, *MLH1*, *MRE11A*, *NBN*, *PALB2*, or *RAD51C* who progressed despite androgen-receptor-targeted therapy and taxane-based chemotherapy [54]. Preliminary data for 43 patients (20 *BRCA1/2*, 14 *ATM*, 2 *PALB2*, 7 other) showed an overall response rate of 25.6% (13.5–41.2), with an ORR of 50% (27.2–72.8) in the *BRCA1/2* subgroup and 7.1% (0.2–33.9) in the *ATM* subgroup.

## 6. Looking into the Future: Potential Biomarkers of Response to PARP Inhibitors, Cautious Optimism, and Ongoing Clinical Trials

*BRCA1* and *BRCA2* are logical candidates to be biomarkers of response to PARPis, based on the current knowledge of DNA damage repair with wild-type BRCA1 and BRCA2 part of the complex in homologous recombination to fix double-strand breaks [55]. However, recent data have sparked debate over just how predictive *BRCA1/2*-mutated cancers can be to PARPis, especially in non-*BRCA*-associated cancer types (cancers not including breast, ovary, prostate, or pancreatic cancer) [56]. Response to PARPis in different *BRCA1/2*-associated cancers varies widely, and *BRCA1/2*-mutations are not synonymous with HRR deficiency, as other secondary somatic mutations may restore or bypass BRCA function [56,57].

Cyclin-dependent kinase 12 (CDK12) phosphorylates the C-terminal domain of RNA polymerase II, which ensures several functions, including optimal transcription elongation, translation of a subset of human protein-coding genes, and maintenance of genomic stability [58]. A genome-wide synthetic lethal screen involving ovarian cancer cell lines and olaparib determined that *CDK12* deficiency may confer sensitivity to PARPis [59]. However, as more clinical data become available, *CDK12* mutations in prostate cancer appear to minimally respond to PARPis. In the TRITON2 trial, *CDK12* mutated mCRPC patients had dismal response rates to rucaparib (0/10 with a radiographic response, 1/15 with a PSA response), and this cohort was discontinued [60]. Antonarakis et al. [61] revealed early results of a multi-institution retrospective study of 60 men with *CDK12*-altered prostate cancers that showed 0 of the 11 who received PARPis (10 olaparib, 1 rucaparib) had a PSA response.

ATM (ataxia-telangiectasia mutated) is a phosphatidylinositol-3 related kinase involved in DNA double-strand break repair that generates signaling networks for DNA repair proteins [62]. Response rates to PARPis in ATM-deficient tumor cell lines were seen in chronic lymphocytic leukemia [28], gastric cancer [63], and mantle cell lymphoma [64]. However, other studies revealed ATM deficiency may not be enough to fully sensitize these cells to PARP inhibition; experiments showed olaparib given alone to ATM-deficient cancer cells induced only a cytostatic state, while there are emerging data suggesting that olaparib combined with an ATR (ATM- and RAD3-related) inhibitor provides an additional cytotoxic effect [65,66]. A study using prostate cancer cell lines agreed that *ATM* loss may not respond to PARPis, but they did respond well to an ATR inhibitor [67].

In the PROFOUND trial, the hazard ratio for progression or death of mCRPC patients with *ATM* mutation was 1.04 (95% CI of 0.61–1.87) [45]. In the TRITON2 trial, only 2 of 19 mCRPC patients with *ATM* mutation receiving rucaparib had a radiographic response and 2 of 49 patients had a PSA response [60]. Taken together, cancers with *ATM* aberrations may rarely respond to PARPis, and the response is, in general, more limited compared with the activity in *BRCA1/2* tumors.

Other DNA repair genes such as *PALB2*, *FANCA* and the *RAD51* family are currently being evaluated in several of the above-mentioned trials, although the number of patients with these mutations enrolled is relatively low. Preclinical data using cell lines have suggested synergy of PARPis with these impaired DNA repair proteins, such as FANCA [68], RAD51C [69], or MRE11 [70]. Limited clinical data exist to make definitive conclusions about the effectiveness of PARPis in patients with these mutations [71]. Notably, prostate cancer patients with *PALB2* mutations receiving different PARPis have demonstrated antitumor activity. More robust prospective studies must be done to better determine the reliability of these biomarkers for PARPis in mCRPC.

Overall success of PARPis in mCRPC must still be interpreted cautiously, as the response to patients harboring various mutations is still variable, and eventually patients experience disease progression after prolonged administration of PARPis. Multiple proposed mechanisms of PARPi resistance include restoration of HRR, DNA replication fork protection, reversion mutations, epigenetic modifications, and restoration of PARylation [72]. Resistance to PARPis was already proposed and demonstrated very early in the preclinical setting using a PARPi-resistant pancreatic cancer cell line with the intragenic deletion of c.6174delT of *BRCA2* [73]. Because of these possibilities, identifying patients with mCRPC who may develop resistance to PARPis would be helpful. For example, Quigley et al. [74] detected *BRCA2* reversion mutations associated with olaparib and talazoparib resistance in mCRPC patients through analysis of circulating cell-free DNA. There continue to be several active clinical trials for mCRPC patients at various stages involving different PARPis, either as monotherapy (Table 1) or combined with other therapies to enhance success (Table 2). These trials aim to further improve upon the progress so far with PARPis against this incurable malignancy.

## 7. Conclusions

The success of PARPis in treating cancer points to the importance of understanding the molecular phenotype of mCRPC, the therapeutic implications of genomic information, and the potential of precision oncology. Olaparib and rucaparib are now available for mCRPC, and other PARPis are likely to be approved soon, based on several ongoing studies. More studies are required to determine the full benefit of these agents, including use in earlier stages of the disease, identification of further predictive biomarkers and evaluation of synergism when combined with other agents.

## Figures and Tables

**Table 1:** Active, recruiting, and planned trials involving castration resistant metastatic prostate cancer and PARP inhibitors as monotherapy.

Trial Number	Notable Characteristics	Phase	Intervention	Primary Outcome Measures
**Olaparib**				
NCT03263650	Aggressive variant prostate cancer Prior cabazitaxel, carboplatin, and prednisone	II	Olaparib	PFS
NCT03434158	mCRPC with HRR defects Prior docetaxel	II	Olaparib	Radiographic PFS
NCT02987543 (PROfound)	mCRPC with HRR defects Prior abiraterone or enzalutamide	III	Olaparib versus enzalutamide or abiraterone	Change in radiographic PFS
**Rucaparib**				
NCT02952534 (TRITON2)	mCRPC with HRR deficiency	II	Rucaparib	ORR and PSA response
NCT03442556	mCRPC with no prior platinum chemotherapy	II	Rucaparib maintenance after induction carboplatin and docetaxel	Radiographic PFS
NCT04171700	Advanced prostate cancer with HRR deficiency besides *BRCA1/2*	II	Rucaparib	Overall response rate
NCT02975934 (TRITON3)	mCRPC with HRR deficiency	III	Rucaparib versus abiraterone, enzalutamide, or docetaxel	Radiographic PFS
**Niraparib**				
NCT02854436 (GALAHAD)	mCRPC Prior taxane and androgen receptor-targeted therapy	II	Niraparib	Objective response rate
NCT04288687	mCRPC Prior platinum-based chemotherapy	II	Niraparib	Radiographic PFS
**Talazoparib**				
NCT03148795 (TALAPRO-1)	mCRPC with HRR deficiency Prior taxane and novel hormonal therapy	II	Talazoparib	Objective response rate

Note: HRR: homologous recombination repair; mCRPC: metastatic castration-resistant prostate cancer; PFS: progression-free survival; PSA: prostate-specific antigen.

**Table 2:** Active, recruiting, and planned trials involving castration resistant metastatic prostate cancer and PARP inhibitors in combination with other agents.

Trial Number	Notable Characteristics	Phase	Intervention	Primary Outcome Measures
**Olaparib**				
NCT02861573	mCRPC	I	Olaparib with pembrolizumab in one cohort; total of four cohorts with all cohorts receiving pembrolizumab	PSA response, adverse events, objective response rate
NCT03205176	mCRPC	I	Olaparib with AZD513 (reversible BRD4 inhibitor)	Dose-limiting toxicity
NCT03874884	mCRPC Prior abiraterone and/or enzalutamide	I	Olaparib with 177Lutetium-prostate- specific membrane antigen	Dose-limiting toxicity, maximum-tolerated dose
NCT02484404	mCRPC Prior enzalutamide/abiraterone or chemotherapy containing docetaxel	I/II	Olaparib with cediranib and MEDI4736 (PD-L1 inhibitor) in different combinations	Safety, overall response rate
NCT02769962	mCRPC Prior enzalutamide or abiraterone or chemotherapy containing docetaxel	I/II	Olaparib with camptothecin	Overall response rate
NCT03317392	mCRPC with metastases to the bone	I/II	Olaparib with radium Ra 223 dichloride	Maximum tolerated dose and radiographic PFS
NCT04556617	mCRPC with homologous recombination repair defects	I/II	Olaparib with PLX2853 (BRD4 inhibitor)	Disease response, dose-limiting toxicities, treatment emergent adverse events
NCT01972217	mCRPC Prior chemotherapy containing docetaxel	II	Olaparib with abiraterone	Safety and tolerability, median radiographic PFS
NCT02893917	mCRPCPrevious taxane therapy	II	Olaparib with cediranib versus olaparib alone	Radiographic PFS
NCT03012321	mCRPC with DNA damage repair defects and no prior chemotherapy or new hormonal agents	II	Abiraterone versus olaparib versus abiraterone with olaparib	Objective PFS
NCT03516812	mCRPC Prior abiraterone and/or enzalutamide	II	Olaparib with testosterone	PSA response
NCT03787680	mCRPC	II	Olaparib with AZD6738 (ATR inhibitor)	Change in radiographic response or PSA
NCT03732820	mCRPC with no prior cytotoxic chemotherapy or new hormonal agents	III	Olaparib with abiraterone versus placebo with abiraterone	Radiographic PFS
NCT03834519	mCRPC Prior abiraterone or enzalutamide, and docetaxel	III	Olaparib with pembrolizumab versus abiraterone versus enzalutamide	Overall survival, radiographic PFS
**Rucaparib**				
NCT04179396	mCRPC	I	Rucaparib with enzalutamide or rucaparib with abiraterone	Pharmacokinetics, adverse events
NCT03338790	mCRPC	I/II	Rucaparib with nivolumab in one cohort; total of three cohorts all receiving nivolumab	ORR, PSA response rate
NCT03572478	mCRPC Prior abiraterone or enzalutamide	I/II	Rucaparib with nivolumab, in combination and as monotherapies	Dose-limiting toxicity, T cell inflammation in the tumor
NCT03840200	mCRPC Prior second-generation androgen receptor targeted therapy	I/II	Rucaparib with ipatasertib	PSA response, dose-limiting toxicity, maximum-tolerated dose, adverse events
NCT04253262	mCRPC Prior abiraterone, enzalutamide, and/or apalutamide	I/II	Rucaparib with copanlisib	Maximum-tolerated dose, response
NCT04455750	mCRPC with no prior therapy while in mCRPC state	III	Rucaparib with enzalutamide	Radiographic PFS, overall survival
**Niraparib**				
NCT03076203	mCRPC At least one prior line androgen receptor-targeted therapy or androgen biosynthesis inhibitor	I	Niraparib with radium Ra 223 dichloride	Maximum-tolerated dose
NCT03431350	mCRPC One or two previous lines of novel androgen receptor-targeted therapy	I/II	Niraparib with cetrelimab or with abiraterone	Toxicity, objective response rate, adverse events, pharmacokinetics
NCT03748641	mCRPC with no prior systemic therapy in the mCRPC setting	III	Niraparib with abiraterone versus placebo with abiraterone	Radiographic PFS
**Talazoparib**				
NCT04019327	mCRPC Progression on at least one second generation hormonal agent	I/II	Talazoparib with temozolomide	Adverse events, overall response rates
NCT03330405	mCRPC with *BRCA* or *ATM* gene defect	I/II	Talazoparib with avelumab	Dose-limiting toxicity, overall response
NCT04052204	mCRPC with DNA damage response defects	I/II	Talazoparib with avelumab and bempegaldesleukin	Dose-limiting toxicity, soft tissue response
NCT03395197 (TALAPRO-2)	mCRPC without prior systemic treatment	III	Talazoparib with enzalutamide versus placebo with enzalutamide	Radiographic PFS
**Veliparib**				
NCT01576172	mCRPC Up to two prior chemotherapy regimens	II	Veliparib with abiraterone versus abiraterone alone	PSA response

Note: mCRPC: metastatic castration-resistant prostate cancer; PARP: poly (ADP-ribose) polymerase; PFS: progression-free survival; PSA: prostate-specific antigen.
